# Comparison of Proteomic Technologies for Blood-Based Detection of Colorectal Cancer

**DOI:** 10.3390/ijms22031189

**Published:** 2021-01-26

**Authors:** Megha Bhardwaj, Tobias Terzer, Petra Schrotz-King, Hermann Brenner

**Affiliations:** 1 Division of Preventive Oncology, German Cancer Research Center (DKFZ) and National Center for Tumor Diseases (NCT), 69120 Heidelberg, Germany; petra.schrotz-king@nct-heidelberg.de (P.S.-K.); h.brenner@dkfz.de (H.B.); 2 Division of Clinical Epidemiology and Aging Research, German Cancer Research Center (DKFZ), 69120 Heidelberg, Germany; 3 Division of Biostatistics, German Cancer Research Center (DKFZ), 69120 Heidelberg, Germany; t.terzer@dkfz-heidelberg.de; 4 German Cancer Consortium (DKTK), German Cancer Research Center (DKFZ), 69120 Heidelberg, Germany

**Keywords:** plasma proteins, colorectal cancer, diagnosis, proximity extension assays, LC/MRM-MS, biomarkers, microarray

## Abstract

Blood-based protein biomarkers are increasingly being explored as supplementary or efficient alternatives for population-based screening of colorectal cancer (CRC). The objective of the current study was to compare the diagnostic potential of proteins measured with different proteomic technologies. The concentrations of protein biomarkers were measured using proximity extension assays (PEAs), liquid chromatography/multiple reaction monitoring–mass spectrometry (LC/MRM-MS) and quantibody microarrays (QMAs) in plasma samples of 56 CRC patients and 99 participants free of neoplasms. In another approach, proteins were measured in serum samples of 30 CRC cases and 30 participants free of neoplasm using immunome full-length functional protein arrays (IpAs). From all the measurements, 9, 6, 35 and 14 protein biomarkers overlapped for comparative evaluation of (a) PEA and LC/MRM-MS, (b) PEA and QMA, (c) PEA and IpA, and (d) LC/MRM-MS and IpA measurements, respectively. Correlation analysis was performed, along with calculation of the area under the curve (AUC) for assessing the diagnostic potential of each biomarker. DeLong’s test was performed to assess the differences in AUC. Evaluation of the nine biomarkers measured with PEA and LC/MRM-MS displayed correlation coefficients >+0.6, similar AUCs and DeLong’s *p*-values indicating no differences in AUCs for biomarkers like insulin-like growth factor binding protein 2 (IGFBP2), matrix metalloproteinase 9 (MMP9) and serum paraoxonase lactonase 3 (PON3). Comparing six proteins measured with PEA and QMA showed good correlation and similar diagnostic performance for only one protein, growth differentiation factor 15 (GDF15). The comparison of 35 proteins measured with IpA and PEA and 14 proteins analyzed with IpA and LC/MRM-MS revealed poor concordance and comparatively better AUCs when measured with PEA and LC/MRM-MS. The comparison of different proteomic technologies suggests the superior performance of novel technologies like PEA and LC/MRM-MS over the assessed array-based technologies in blood-protein-based early detection of CRC.

## 1. Introduction

Colorectal cancer (CRC) is the third most common cancer globally, accounting for approximately 1.85 million incident cases and 880,000 deaths per year [1]. In recent years, a large number of studies have evaluated the potential of minimally invasive blood-based DNA [2,3], RNA [4,5], metabolite [6] and protein [7] biomarkers for CRC diagnosis. Since the proteins undergo dynamic changes in response to any disease and serve as functional link between genomes and phenotypes, protein biomarkers from plasma or serum could be potentially useful as a screening tool for early detection of CRC. Utilizing novel high-throughput approaches that enable simultaneous screening of hundred-plex targets, research studies have specifically explored the blood proteome and identified signatures for early detection of CRC [7,8,9,10,11]. There is a lack of specific validated biomarkers for detecting early stages of CRC [7,10,12]. It has also been established that only the selection of participants from true screening settings, such as participants in screening colonoscopy, rather than clinical settings, would ensure fully comparable recruitment conditions for cases and controls to that of the target screening population [13]. Even though many blood-based protein signatures have been identified and numerous proteomic technologies are available, direct comparisons of the diagnostic performance of the technologies for detection of cancer in screening settings are scarce. Previous studies from our group utilizing proximity extension assays (PEA) and liquid chromatography/multiple reaction monitoring–mass spectrometry (LC/MRM-MS) have identified several individual markers and multimarker protein signatures for the detection of CRC [13,14,15,16,17,18]. The objective of the current study was to evaluate and compare the diagnostic potential of overlapping protein biomarkers measured with PEA, LC/MRM-MS, quantibody microarrays (QMAs) and immunome full-length functional protein arrays (IpAs).

## 2. Results

To identify, evaluate and validate biomarkers for early detection of CRC, protein targets were assayed using different proteomic technologies like PEA, LC/MRM-MS, QMA and IpA. For the current study, the comparisons were established for protein biomarkers that overlapped between measurements from different technologies.

### 2.1. Characteristics of the Study Populations

**Figure 1:**
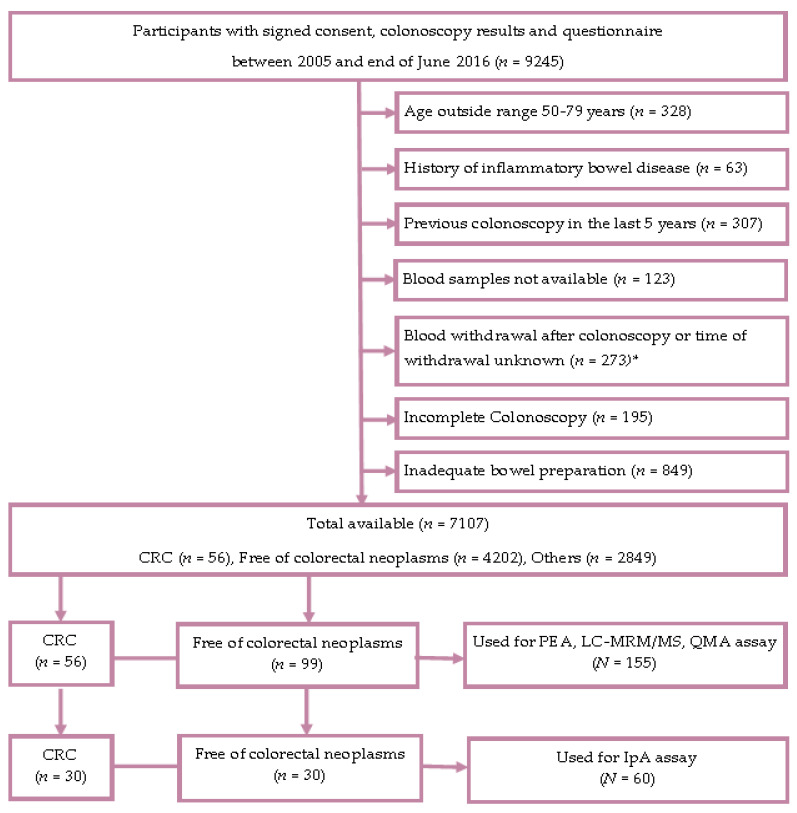
STARD (Standards for the Reporting of Diagnostic Accuracy) flow diagram (BLITZ study). Abbreviations: CRC, colorectal cancer; IpA, immunome protein array; LC/MRM-MS, liquid chromatography/multiple reaction monitoring–mass spectrometry; *n*/*N*, number; SD, standard deviation; PEA, proximity extension assay; QMA, quantibody microarray. * The exclusion criteria for the selection of CRC cases were not applicable after this point.

Figure 1 provides the Standards for Reporting of Diagnostic Accuracy studies (STARD) diagram displaying the selection of study participants enrolled in the Begleitende Evaluierung innovativer Testverfahren zur Darmkrebs-Früherkennung (BLITZ) study.

As shown in Table 1, the PEA, LC/MRM-MS and QMA measurements were performed on 56 and 99 participants with CRC and free of neoplasms, respectively.

The median age of the study participants was around 65 years and 66 years for the cases and controls, respectively, and males represented 64% of participants in both the groups. The study population for IpA assays consisted of 30 participants in screening colonoscopy with CRC and 30 controls free of neoplasms, with a median age of 64.5 years and males representing 60% of the study populations in both the groups.

### 2.2. Assay Performance

More than 97% of the samples met the quality control criteria (QCC) for the plasma protein biomarkers measured with PEA and the average intra-assay coefficient of variance (CV) and average inter-assay CVs were 7% and 9%, respectively. For the LC/MRM-MS and QMA measurements, 99% samples met the QCC, and the CV for both assays was <20%. The serum protein IpA measurements of all the samples met the QCC and the mean CV across all samples was 8%.

### 2.3. Comparison of PEA with LC/MRM-MS

The results of the correlation analysis for the nine protein biomarkers measured in the same plasma samples (CRC cases = 56; controls free of neoplasms = 99) using PEA and LC/MRM-MS assays are summarized in Table 2. Pearson’s product–moment correlation coefficients were ≥0.6 for four biomarkers, i.e., insulin-like growth factor binding protein 2 (IGFBP2), matrix metalloproteinase 9 (MMP9), serum paraoxonase lactonase 3 (PON3) and myeloblastin (PRTN3). Upon application of adjustment for multiple testing, significant differences (at an adjusted *p*-value ≤ 0.05) between CRC cases and controls were found for two and three protein biomarkers using PEA and LC/MRM-MS, respectively.

**Figure 2:**
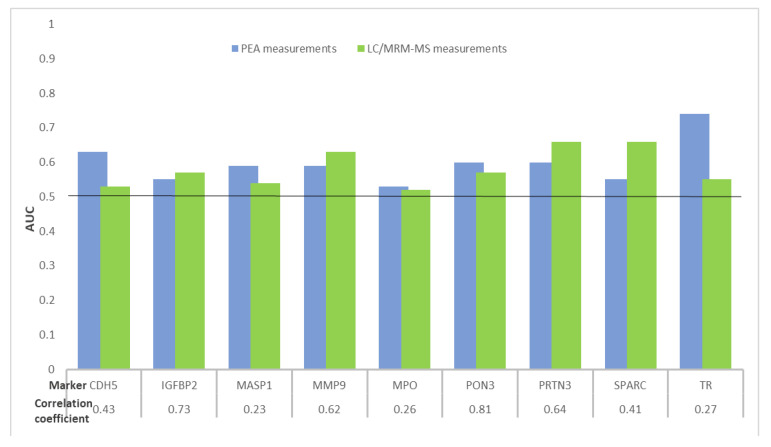
Distribution of AUCs of nine protein biomarkers overlapping between PEA and LC/MRM-MS measurements for detecting CRC cases from participants in screening colonoscopy. Abbreviations: AUC, area under the receiver operating curve; CDH5, cadherin 5; CRC, colorectal cancer; IGFBP2, insulin-like growth factor binding protein 2; LC/MRM-MS, liquid chromatography/multiple reaction monitoring–mass spectrometry; MASP1, mannan binding lectin serine protease 1; MMP9, matrix metalloproteinase 9; MPO, myeloperoxidase; PEA, proximity extension assay; PON3, serum paraoxonase lactonase 3; PRTN3, myeloblastin; SPARC, secreted protein acidic and rich in cysteine; TR, transferrin receptor protein 1.

As shown in Figure 2 and Table 2, areas under the receiver operating curves (ROCs) (AUC) ≥ 0.60 were observed for four and three protein biomarkers using PEA and LC/MRM-MS assays, respectively. Of the nine analyzed proteins, the best individual diagnostic performances were observed for transferrin receptor protein 1 (TR) and secreted protein acidic and rich in cysteine (SPARC) with AUCs and their 95% confidence intervals (95% CIs) of 0.74 (95% CI, 0.65–0.82) and 0.66 (95% CI, 0.58–0.75) for PEA and LC/MRM-MS measurements, respectively. Similar AUCs were observed for the biomarkers IGFBP2, MMP9, myeloperoxidase (MPO) and PON3 measured using both technologies. Only for one of the nine proteins was the AUC significantly different (*p* < 0.05) between the two measurements.

### 2.4. Comparison of PEA with QMA

**Figure 3:**
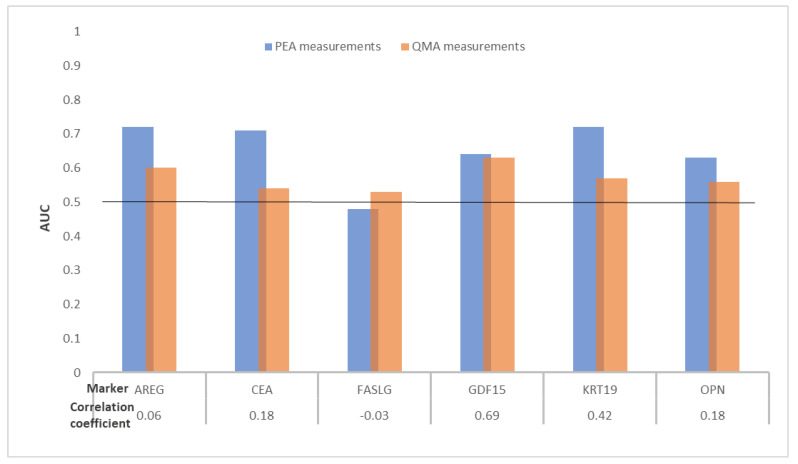
Distribution of AUCs of six protein biomarkers overlapping between PEA and QMA measurements for detecting CRC cases from participants in screening colonoscopy. Abbreviations: AREG, amphiregulin; AUC, area under the receiver operating curve; CEA, carcinoembryonic antigen; CRC, colorectal cancer; FASLG, Fas ligand; GDF15, growth differentiation factor 15; KRT19, keratin Type I cytoskeletal 19; OPN, osteopontin; PEA, proximity extension assay; QMA, quantibody microarray.

The head-to-head comparison of the six proteins measured using PEA and QMA in plasma from 56 CRC cases and 99 controls free of neoplasms is presented in Figure 3 and Table 3.

Good concordance (correlation coefficient = 0.69) was observed for only one protein, growth differentiation factor 15 (GDF15). Adjusted *p*-values ≤ 0.05 were observed for five proteins and one protein using PEA and QMA, respectively. For the six biomarkers measured, AUCs ≥ 0.60 were observed for all six biomarkers measured using PEA and for only two protein biomarkers with QMA. Individually the biomarkers with the best diagnostic potentials were amphiregulin (AREG) and GDF15, with AUCs of 0.72 (95% CI, 0.64–0.81) and 0.63 (95% CI, 0.54–0.72) from PEA and QMA measurements, respectively. The difference in AUCs for the six biomarkers measured using both assays were assessed and DeLong *p*-values ≤ 0.05 were observed for two biomarkers.

### 2.5. Comparison of PEA with IpA

**Figure 4:**
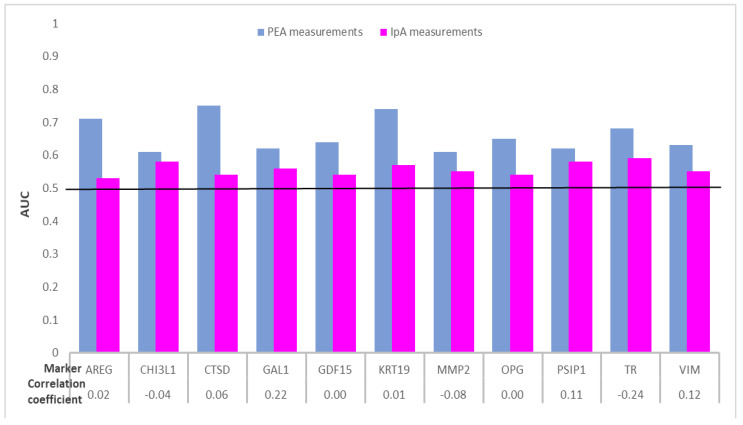
Distribution of AUCs of 11 protein biomarkers overlapping between PEA and IpA measurements for detecting CRC cases from participants in screening colonoscopy. Abbreviations: AREG, amphiregulin; AUC, area under the receiver operating curve; CHI3L1, chitinase-3-like protein 1; CRC, colorectal cancer; CTSD, cathepsin D; GAL1, galectin-1; GDF15, growth differentiation factor 15; IpA, immunome protein array; KRT19, keratin Type I cytoskeletal 19; MMP2, matrix metalloproteinase-2; OPG, osteoprotegerin; PEA, proximity extension assay; PSIP1, PC4 and SFRS1-interacting protein; TR, transferrin receptor protein 1; VIM, vimentin.

Eleven out of the 35 plasma protein biomarkers were identified with AUCs ≥ 0.60 with PEA assays, whereas AUCs were below 0.60 for all serum protein biomarkers using IpA measurements. The comparative results for these 11 biomarkers are presented in Figure 4 and Table 4; the results for the remaining biomarkers are presented in Table S1.

As shown in Table 4, the results of the Pearson’s product–moment correlation analysis for the same protein biomarkers measured in plasma samples using PEA and serum samples using IpA (30 CRC cases and 30 controls) revealed that there was no correlation between the biomarkers measured. When adjusted for multiple testing, *p*-values ≤ 0.05 were observed for two proteins using PEA and none of the biomarkers using IpA. From the proteins measured using PEA, AUCs of 0.75, 0.74 and 0.71 were observed for the proteins cathepsin D (CTSD), keratin Type I cytoskeletal 19 (KRT19) and AREG, respectively. However, none of the IpA-measured biomarkers distinguished cases and controls with AUCs ≥ 0.60.

### 2.6. Comparison of LC/MRM-MS with IpA

**Figure 5:**
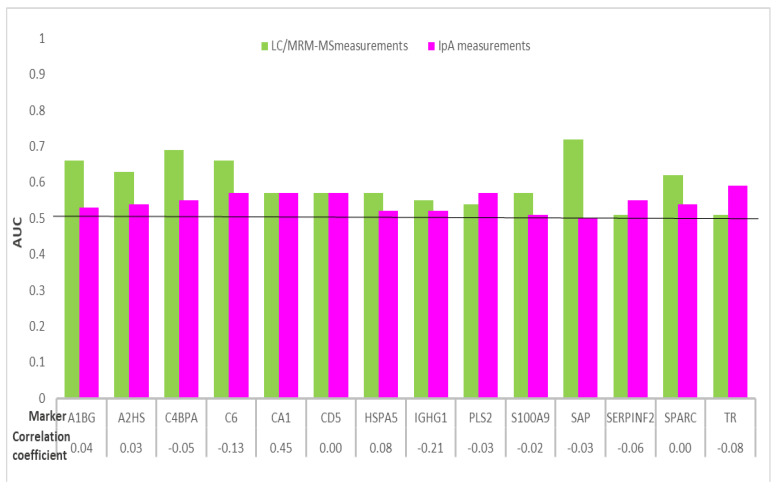
Distribution of AUCs of 14 protein biomarkers overlapping between LC/MRM-MS and IpA measurements for detecting CRC cases from participants in screening colonoscopy. Abbreviations: A1BG, alpha-1B-glycoprotein; A2HS, alpha-2-HS-glycoprotein; AUC, area under the receiver operating curve; C4BPA, C4b, binding protein alpha chain; C6, complement component C6; CA1, carbonic anhydrase 1; CD5, CD5 antigen; CRC, colorectal cancer; HSPA5, 78 kDa glucose-regulated protein; IGHG1, immunoglobulin gamma-1 chain C region; IpA, immunome protein array; LC/MRM-MS, liquid chromatography/multiple reaction monitoring–mass spectrometry; PLS2, plastin-2; S100A9, protein S100-A9; SAP, serum amyloid P-component; SERPINF2, alpha-2-antiplasmin; SPARC, secreted protein acidic and rich in cysteine; TR, transferrin receptor protein 1.

The results of the comparative evaluation for 14 protein biomarkers measured in plasma with LC/MRM-MS and in serum with IpA for samples from 30 CRC cases and 30 controls are presented in Figure 5 and Table 5.

As observed for the PEA and IpA comparison, no correlation was observed for the 14 biomarkers measured with LC/MRM-MS and IpA. Adjusted *p*-values ≤ 0.05 were observed for two proteins with LC/MRM-MS measurements, whereas none of the biomarkers measured with IpA presented with such *p*-values. The AUCs ≥ 0.60 were observed for 6 out of 14 biomarkers measured using LC/MRM-MS and for none of the biomarkers measured with IpA. Individually, the biomarker serum amyloid P component (SAP) measured with LC/MRM-MS best detected CRC cases with an AUC 0.72 (95% CI, 0.58–0.85).

## 3. Discussion

In the current study, the diagnostic potential of protein biomarkers measured with proximity extension assays, liquid chromatography/multiple reaction monitoring–mass spectrometry, quantibody microarray and immunome full-length functional protein array were compared wherever possible for overlapping proteins in blood samples from participants undergoing screening colonoscopy. Overall, the comparison of nine overlapping biomarkers measured with PEA and LC/MRM-MS displayed good concordance and similar diagnostic potential for the biomarkers IGFBP2, MMP9 and PON3. However, when proteins measured with QMA and IpA assays were compared with the PEA measurements, poor concordance and relatively low AUCs were observed even for biomarkers like AREG and KRT19 that have been associated with CRC. Similarly, when biomarkers measured with IpA were compared with the LC/MRM-MS measurements, poor concordance and relatively low AUCs were observed for IpA-measured proteins.

In search for diagnostic biomarkers, in addition to blood, alternative biological substances like urine [19], saliva [20] and exhaled air [21] are currently being explored. However, it has been shown that a wide range of cancer-associated biomarkers are likely to end up in systemic blood circulation, and blood samples can be easily obtained with minimal risk and at nominal costs [22]. Furthermore, blood-based tests are potentially more acceptable for screening of the general population, as they are less invasive compared with screening colonoscopy, which is the existing gold standard for early CRC detection. Therefore, a large number of studies have evaluated the potential of blood-based biomarkers associated with CRC diagnosis [23,24,25,26,27]. Although liquid biopsy with genetic and epigenetic biomarkers is a promising approach, a lack of preanalytical and analytical consensus, inadequate clinical validation and insufficient regulatory endorsements are the challenges that need to be addressed before these markers can be clinically utilized [27,28]. While validated proteomic assays are comparatively standardized and analytically more optimized, future diagnostic research would benefit from a combination of protein and non-protein biomarkers.

An ideal biomarker should be quantitatively measurable, highly specific, extremely sensitive, validated, reliable and reproducible [29,30]. A key factor in this context is also the discriminative power i.e., the diagnostic performance of a biomarker to distinguish between cases and controls. Additionally, validation of early detection markers in screening settings is indispensable because differences between cancer cases and cancer-free controls observed in case–control settings (i.e., in settings in which cases are recruited and blood samples are taken after cancer diagnosis) might reflect post-symptom or even post-diagnostic changes of the proteome [7,9]. The current study hence selected participants from a true prospective screening cohort, with all participants undergoing screening colonoscopy, and compared the potential of proteins identified by different detection and quantitation platforms.

Technologies that enable simultaneous detection of less abundant markers in a low sample volume were compared in the current study. PEA is a unique technology that utilizes a pair of oligonucleotide-labeled antibodies as probes that lead to the initiation of an amplified signal detection when the antibodies bind pairwise in close proximity to each other. This dual recognition of the protein makes PEA a target-sensitive and -specific method. LC/MRM-MS functions by quantitation of selected protein targets based on heavy and light synthetic peptides. The unique identification of each target protein by selected peptides and the use of a triple-quadrupole, allowing only a selected peptide and only a specific fragment to pass through the first and second quadrupoles, makes LC/MRM-MS a target-specific method. The quantibody microarray represents a technique in which the antibody is immobilized on a surface of a glass slide and, when incubated with blood plasma, the antigens from the plasma samples bind to those antibodies, which are later detected by fluorescence. The immunome protein array has an excellent signal-to-noise ratio and every protein spotted on the array is full length, correctly folded and functionally validated. Misfolded proteins lack biotinylation and this ensures that only correctly folded proteins are immobilized on the array. The technical assay sensitivities of PEA, LC/MRM-MS, QMA and IpA are in the low picogram/mL, mid-high nanogram/mL, mid-picogram/mL and picogram/mL ranges, respectively. Sample volumes of 1 µL, 20 µL, 50 µL and 20 µL were used for the PEA, LC/MRM-MS, QMA and IpA assays, respectively.

In a previous study from our group, 11 protein biomarkers assayed using PEA and LC/MRM-MS were compared in plasma samples from clinically recruited CRC cases [16]; in that study, a good correlation was found for 8 out of 11 biomarkers. In the current study, good correlations could be observed for four of the nine biomarkers measured using PEA and LC/MRM-MS in samples from participants of screening colonoscopy. Comparison of PEA with QMA measurements showed good correlation for only one out of six protein biomarkers. Evaluation of 35 overlapping biomarkers from PEA and IpA measurements and 14 proteins overlapping from LC/MRM-MS and IpA measurements revealed poor concordance for all biomarkers. Although our results suggest the inferiority of the array-based technologies compared with PEA and LC/MRM-MS measurements in diagnostic CRC biomarker research, array-based platforms may potentially be useful for exploring protein interactions or alterations.

To our knowledge, this is the first study to evaluate the diagnostic potential of protein biomarkers using different proteomic technologies in participants in screening colonoscopies. The four technologies used in the current study possess the ability to detect less abundant markers using low volumes of blood. The samples in the current study were selected from screening participants to obtain results that would be generalizable to the target screening population. Since identical samples were selected for different assay measurements, any pre-analytical bias arising due to differences in sample handling, collection and storage were avoided. A major limitation is the relatively small number of CRC cases, despite the very large screening population they were derived from. The number of CRC cases was further restricted for IpA measurements because of the very high cost of the platform. The set of proteins assessed was limited by their pairwise joint availability and inclusion in the different platforms. However, despite these limitations, differences in the diagnostic performance of the protein biomarkers could clearly be demonstrated and this important information may help in guiding future research in blood-based protein diagnostic biomarker research.

## 4. Materials and Methods

### 4.1. Study Population

For the assessment of proteins, the blood samples were selected from participants in screening colonoscopy collected in the study. Details of the BLITZ study design have been reported previously [14,31,32,33,34,35]. In brief, BLITZ is an ongoing screening study of participants in the German screening colonoscopy program offered to men and women aged 55 and older since 2002. Participants have been recruited from 20 gastroenterology practices in southern Germany since end of 2005. By the end of June 2016, 9245 participants had been recruited, of whom 2138 were excluded based on the selection criteria displayed in the STARD diagram presented in Figure 1. Among the remaining 7107 participants, CRC had been detected in 56 participants. In the current study for the detection of different proteins with PEA, LC/MRM-MS and QMA, plasma samples from 56 participants with CRC and 99 controls free of colorectal neoplasms were used. For detecting proteins with IpA serum, samples from 30 participants with CRC and 30 controls free of neoplasms were selected. In both instances, participants free of colorectal neoplasm were frequency-matched to CRC by age and sex. Use of samples from the BLITZ study for the evaluation of early detection markers for CRC has been approved by the ethics committees of the Medical Faculty Heidelberg (S-178/2005, 2005) and of the physicians’ boards of Baden-Wuerttemberg (M118-05-f, 2011), Rhineland-Palatinate (837.047.06(5145), 2017), Hesse (MC 254/2007, 2007) and Saarland (217/13, 2013).

### 4.2. Sample Collection and Storage

Blood samples were collected before colonoscopy, i.e., before the first diagnosis of CRC and before any therapeutic intervention. After blood draw, plasma and serum samples were transported to the laboratory while being preserved in a cold transport chain, followed by centrifugation at 2000–2500× *g* for 10 min at 4 °C, aliquoted and then stored at −80 °C until picked out for the protein measurements.

### 4.3. Laboratory Assays

Using four multiplex protein detection and quantitation methods like PEA, LC/MRM-MS, QMA and IpA, different sets of protein targets were assayed. The sets of proteins were not selected by the authors but analyzed as commercially provided by different manufacturers.

The concentrations of proteins in plasma samples were measured firstly by PEA offered by Olink, Uppsala, Sweden [36]. Multiplex panels from Olink allowed simultaneous analysis of 92 biomarkers in 1 µL samples; the full protocol of the PEA has been reported previously [37]. Briefly, 96 pairs of oligonucleotide-labeled antibodies (92 biomarkers and 4 internal controls) were allowed to bind pairwise to target proteins and, when they were in close proximity, a PCR reporter sequence was formed due to DNA polymerization, which was quantified by real-time PCR. In total, 275 proteins were analyzed using the “Oncology II”, “Immune response” and “Cardiovascular III” Olink multiplex panels [17]; the list of proteins in the different panels is available from the manufacturer’s website [38].

In another approach, plasma samples were also analyzed using LC/MRM-MS for the targeted quantitation by peptide-based analysis for 270 unique proteins [18]. The LC/MRM-MS assays were performed at the Genome British Columbia Proteomics Centre, University of Victoria, Victoria, British Columbia, Canada [39]; the list of proteins can be found here [40]. The full protocol of LC/MRM-MS has been published elsewhere [41,42]; in brief, the quantitation of the proteins was performed using paired heavy and light synthetic peptides for every protein. The unlabeled light peptides were used to create the calibration curves and the heavy stable isotope-labeled internal standard peptide was spiked into every plasma sample at a fixed, predetermined concentration. LC/MRM-MS is highly reproducible and has been previously validated by following the Clinical Proteome Tumor Analysis Consortium guidelines for assay development [43].

The plasma samples were analyzed for 11 other proteins in parallel using a customized QMA (Quantibody G-Series Human TH17 Array 1) provided by RayBiotech, Norcross, GA, USA [44]. Each QMA was spotted with 16 wells of identical antibodies for 11 targets, and each antibody was spotted in quadruplicate. Each array was spotted with a sandwich-based matched pair of target-specific antibodies and the streptavidin–biotin fluorescence readout was used for detection.

Lastly, the measurement of cancer-related proteins was performed in serum samples from 60 participants using the IpA offered by Sengenics, Singapore, Republic of Singapore. The IpA allows multiplex analysis of 1627 full-length human proteins from biologically significant families including kinases, signaling molecules, cytokines, interleukins, chemokines and cancer antigens, and has been described formerly [45]; the list of all targets is available on the manufacturer’s website [46]. In brief, all proteins on the IpA were correctly folded and functional as they were expressed as fusion proteins with biotin carboxyl carrier protein (BCCP), which acted as a folding marker and solubility enhancer. The arrays were coated with a proprietary surface that preserved protein folding and inhibited binding of non-specific proteins. Interaction between antigens was detected as fluorescence of the bound fluorescence-labeled IgG, and each antigen was immobilized in quadruplicate on the array.

Out of all the targets measured across 4 platforms, (a) 9 proteins overlapped between the PEA and LC/MRM-MS measurements, (b) 6 proteins overlapped between the PEA and QMA assays, (c) 35 proteins overlapped between the PEA and IpA measurements and (d) 14 proteins overlapped between the LC/MRM-MS and IpA measurements; the current research focused on these overlapping biomarkers. All laboratory analyses were performed blind with respect to the findings at colonoscopy.

### 4.4. Statistical Analysis

The linear plasma protein values from PEA were log-transformed to produce the normalized protein expression (NPX). NPX represents the relative signal on a log_2_ scale and one unit of NPX difference represents a 2-fold change in protein concentration. The LC/MRM-MS protein values were visualized and examined with Skyline Quantitative Analysis software (version 4.1.1.18179, MacCoss Lab, Department of Genome, University of Washington, Seattle, WA, USA). With defining criteria such as 1/*x* regression weighting and < 20% deviation in the accuracy of the quality control sample, the standard curve was used to calculate the peptide concentrations for each protein. For the QMA assays, the median of quadruplicate fluorescence intensities for each protein was taken as the value for that protein. The intra- and inter-assay normalization was performed on these values and the linear data were log-transformed in order to make them comparable with the protein values from other assays. For the serum protein measurements using IpA, each protein and control probe was spotted in quadruplicate, and the foreground and background intensities of each spot was represented in relative fluorescence units (RFUs). The median of these four spot readings was taken as the final value, and both quantile-based and total intensity normalization of the data were performed [47]. The linear serum protein RFU values obtained from the IpA were log-transformed.

As no single protein biomarker was measured using all 4 technologies, the head-to-head comparisons were established for 9, 6, 35 and 14 protein biomarkers that overlapped between PEA vs. LC/MRM-MS, PEA vs. QMA, PEA vs. IpA and LC/MRM-MS vs. IpA measurements, respectively. For these 4 aforementioned comparisons, Pearson’s product–moment correlation was calculated to measure linear relationships between the concentration values of proteins. Subsequently, in order to evaluate the diagnostic performance for distinguishing CRC cases from controls free of neoplasms, Mann–Whitney tests [48] with adjustment for multiple testing by the Benjamini and Hochberg method were performed [49]. For each individual protein biomarker measured across different platforms, a logistic regression model was used to construct the prediction algorithm [50], and diagnostic performance indicators including AUCs (95% CI) and estimates of sensitivity at cutoffs yielding 80% and 90% specificity were calculated. The DeLong test was performed to assess differences between the AUCs obtained for the same biomarker measured using 2 different assays, respectively [51]. All statistical analyses were performed with statistical software R language and environment, Vienna, Austria (version 3.6.3, R core team) [52] using R packages ‘dplyr’, ‘glmnet’, ‘ModelGood’, and ‘pROC’. Statistical testing was two-sided, and *p*-values of 0.05 or less were considered to be statistically significant.

## 5. Conclusions

Investigating the proteins with different competitive target-specific detection methods, we evaluated the diagnostic performance of blood-based protein biomarkers individually for the detection of CRC. The comparison of different proteomic technologies suggests the superior performance of novel technologies like PEA and LC/MRM-MS over the assessed array-based technologies in blood-protein-based early detection of CRC.

## Figures and Tables

**Table 1:** Characteristics of the study population from Begleitende Evaluierung innovativer Testverfahren zur Darmkrebs-Früherkennung (BLITZ) participants in screening colonoscopy.

Group	BLITZ (Screening)			
	PEA, LC/MRM-MS and QMA Assays		IpA Assays	
Total	CRC	Controls	CRC	Controls
	*N* (%)	*N* (%)	*N* (%)	*N* (%)
	56	99	30	30
Age in years				
50–59	10 (18)	20 (20)	5 (16)	5 (16)
60–69	28 (50)	49 (50)	14 (47)	14 (47)
70–79	18 (32)	30 (30)	11 (37)	11 (37)
Mean	66.0	65.4	66.2	66.2
Median	65.0	66.0	64.5	64.5
SD	5.8	6.9	6.9	6.9
Gender distribution				
Male	36 (64)	63 (64)	18 (60)	18 (60)
Female	20 (36)	36 (36)	12 (40)	12 (40)
Stage distribution				
Stage I	17 (30)	-	9 (30)	-
Stage II	6 (11)	-	6 (20)	-
Stage III	26 (46)	-	8 (27)	-
Stage IV	7 (13)	-	7 (23)	-
Early stage (I/II)	23 (41)	-	15 (50)	-
Late stage (III/IV)	33 (59)	-	15 (50)	-

Abbreviations: CRC, colorectal cancer; IpA, immunome protein array; LC/MRM-MS, liquid chromatography/multiple reaction monitoring–mass spectrometry; *N*, number; SD, standard deviation; PEA, proximity extension assay; QMA, quantibody microarray.

**Table 2:** Diagnostic performance of nine biomarkers overlapping between PEA and LC/MRM-MS measurements for detecting CRC cases from participants in screening colonoscopy

Marker	Pearson’s Correlation Coefficient	PEA Measurements					LC/MRM-MS Measurements					DeLong *p*-value for AUC Testing
		AUC (95% CI)	*p*-value	*p*-value ^adj^	Se% at 80% Sp	Se% at 90% Sp	AUC (95% CI)	*p*-value	*p*-value ^adj^	Se% at 80% Sp	Se% at 90% Sp	
CDH5	0.43	0.63 (0.54–0.72)	<0.01 **	<0.05 *	32	11	0.53 (0.43–0.62)	0.55	0.61	21	16	0.06
IGFBP2	0.73	0.55 (0.45–0.65)	0.31	0.38	30	23	0.57 (0.47–0.66)	0.17	0.31	32	23	0.49
MASP1	0.23	0.59 (0.49–0.69)	0.06	0.11	30	25	0.54 (0.45–0.64)	0.35	0.44	27	16	0.55
MMP9	0.62	0.59 (0.49–0.68)	0.08	0.11	38	23	0.63 (0.54–0.72)	<0.01 **	<0.05 *	36	25	0.29
MPO	0.26	0.53 (0.44–0.62)	0.56	0.56	14	11	0.52 (0.44–0.60)	0.61	0.61	21	16	0.87
PON3	0.81	0.60 (0.51–0.69)	<0.05 *	0.09	27	11	0.57 (0.48–0.67)	0.13	0.31	23	16	0.35
PRTN3	0.64	0.60 (0.51–0.69)	<0.05 *	0.09	13	9	0.66 (0.58–0.75)	<0.01 **	<0.01 **	41	16	0.15
SPARC	0.41	0.55 (0.45–0.64)	0.34	0.38	18	11	0.66 (0.58–0.75)	<0.01 **	<0.01 **	45	25	0.14
TR	0.27	0.74 (0.65–0.82)	<0.01 **	<0.01 **	52	39	0.55 (0.45–0.65)	0.34	0.44	32	25	<0.05 *

Abbreviations: AUC, area under the receiver operating curve; CRC, colorectal cancer; 95% CI, 95% confidence interval; LC/MRM-MS, liquid chromatography/multiple reaction monitoring–mass spectrometry; PEA, proximity extension assay; *p*-value, apparent *p*-values without any adjustments; *p*-value ^adj^, *p*-value after adjustment for multiple testing by the Benjamini–Hochberg method; Se, sensitivity; Sp, specificity. Protein abbreviations: CDH5, cadherin 5; IGFBP2, insulin-like growth factor binding protein 2; MASP1, mannan binding lectin serine protease 1; MMP9, matrix metalloproteinase 9; MPO, myeloperoxidase; PON3, serum paraoxonase lactonase 3; PRTN3, myeloblastin; SPARC, secreted protein acidic and rich in cysteine; TR, transferrin receptor protein 1. Note: *, Significant at a *p*-value of < 0.05; **, significant at a *p*-value of < 0.01.

**Table 3:** Diagnostic performance of six biomarkers overlapping between PEA and QMA measurements for detecting CRC cases from participants in screening colonoscopy.

Marker	Pearson’s Correlation Coefficient	PEA Measurements					QMA Measurements					DeLong *p*-value for AUC Testing
		AUC (95% CI)	*p*-value	*p*-value ^adj^	Se% at 80% Sp	Se% at 90% Sp	AUC (95% CI)	*p*-value	*p*-value ^adj^	Se% at 80% Sp	Se% at 90% Sp	
AREG	0.06	0.72 (0.64–0.81)	<0.01 **	<0.01 **	57	39	0.60 (0.51–0.70)	<0.05 *	0.10	32	20	0.06
CEA	0.18	0.71 (0.62–0.80)	<0.01 **	<0.01 **	63	43	0.54 (0.45–0.64)	0.38	0.46	27	20	<0.05 *
FASLG	−0.03	0.48 (0.39–0.58)	0.75	0.75	27	20	0.53 (0.44–0.62)	0.55	0.55	20	13	0.49
GDF15	0.69	0.64 (0.55–0.73)	<0.01 **	<0.05 *	34	27	0.63 (0.54–0.72)	<0.05 *	<0.05 *	41	16	0.84
KRT19	0.42	0.72 (0.63–0.80)	<0.01 **	<0.01 **	52	38	0.57 (0.47–0.66)	0.16	0.31	29	20	<0.05 *
OPN	0.18	0.63 (0.54–0.72)	<0.01 **	<0.05 *	38	23	0.56 (0.47–0.66)	0.20	0.31	27	13	0.25

Abbreviations: AUC, area under the receiver operating curve; CRC, colorectal cancer; 95% CI, 95% confidence interval; PEA, proximity extension assay; *p*-value, apparent *p*-values without any adjustments; *p*-value ^adj^, *p*-value after adjustment for multiple testing by the Benjamini–Hochberg method; QMA, quantibody microarray; Se, sensitivity; Sp, specificity. Protein abbreviations: AREG, amphiregulin; CEA, carcinoembryonic antigen; FASLG, Fas ligand; GDF15, growth differentiation factor 15; KRT19, keratin Type I cytoskeletal 19; OPN, osteopontin. Note: *, Significant at a *p*-value of < 0.05; **, significant at a *p*-value of < 0.01.

**Table 4:** Diagnostic performance for 11 out of 35 biomarkers overlapping from PEA and IpA measurements for detecting CRC cases from participants in screening colonoscopy.

Marker	Pearson’s Correlation Coefficient	PEA Measurements					IpA Measurements					DeLong *p*-value for AUC Testing
		AUC (95% CI)	*p*-value	*p*-value ^adj^	Se % at 80% Sp	Se % at 90% Sp	AUC (95% CI)	*p*-value	*p*-value ^adj^	Se % at 80% Sp	Se % at 90% Sp	
AREG	0.02	0.71 (0.57–0.84)	<0.01 **	0.07	45	28	0.53 (0.38–0.68)	0.71	0.85	19	10	0.09
CHI3L1	−0.04	0.61 (0.46–0.76)	0.15	0.56	26	17	0.58 (0.44–0.73)	0.27	0.79	15	7	0.07
CTSD	0.06	0.75 (0.63–0.88)	<0.01 **	<0.05 *	55	35	0.54 (0.39–0.69)	0.61	0.79	14	6	<0.01 **
GAL1	0.22	0.62 (0.48–0.77)	0.11	0.53	23	9	0.56 (0.41–0.71)	0.43	0.79	13	6	0.56
GDF15	0.00	0.64 (0.50–0.79)	0.06	0.40	31	18	0.54 (0.39–0.69)	0.56	0.79	14	6	0.08
KRT19	0.01	0.74 (0.61–0.86)	<0.01 **	<0.05 *	47	37	0.57 (0.42–0.72)	0.34	0.79	15	9	0.10
MMP2	−0.08	0.61 (0.46–0.75)	0.15	0.56	23	12	0.55 (0.40–0.70)	0.51	0.79	13	6	0.14
OPG	0.00	0.65 (0.51–0.80)	<0.05 *	0.33	41	32	0.54 (0.39–0.69)	0.63	0.79	16	8	0.07
PSIP1	0.11	0.62 (0.47–0.76)	0.12	0.53	18	7	0.58 (0.43–0.73)	0.31	0.79	17	8	0.07
TR	−0.24	0.68 (0.55–0.82)	<0.01 **	0.14	33	26	0.59 (0.45–0.74)	0.22	0.79	15	7	<0.01 **
VIM	0.12	0.63 (0.49–0.78)	0.08	0.47	28	10	0.55 (0.40–0.70)	0.49	0.81	36	13	0.45

Abbreviations: AUC, area under the receiver operating curve; CRC, colorectal cancer; 95% CI, 95% confidence interval; IpA, immunome protein array; PEA, proximity extension assay; *p*-value, apparent *p*-values without any adjustments; *p*-value ^adj^, *p*-value after adjustment for multiple testing by the Benjamini–Hochberg method; Se, sensitivity; Sp, specificity. Protein abbreviations: AREG, amphiregulin; CHI3L1, chitinase-3-like protein 1; CTSD, cathepsin D; GAL1, galectin-1; GDF15, growth differentiation factor 15; KRT19, keratin Type I cytoskeletal 19; MMP2, matrix metalloproteinase-2; OPG, osteoprotegerin; PSIP1, PC4 and SFRS1-interacting protein; TR, transferrin receptor protein 1; VIM, vimentin. Note: *, Significant at a *p*-value of < 0.05; **, significant at a *p*-value of < 0.01.

**Table 5:** Diagnostic performance of 14 biomarkers overlapping between LC/MRM-MS and IpA measurements for detecting CRC cases from participants in screening colonoscopy.

Marker	Pearson’s Correlation Coefficient	LC/MRM-MS Measurements					IpA Measurements					DeLong *p*-value for AUC Testing
		AUC (95% CI)	*p*-value	*p*-value ^adj^	Se % at 80% Sp	Se % at 90% Sp	AUC (95% CI)	*p*-value	*p*-value ^adj^	Se % at 80% Sp	Se % at 90% Sp	
A1BG	0.04	0.66 (0.51–0.80)	<0.05 *	0.15	45	34	0.53 (0.38–0.69)	0.69	0.80	17	10	0.26
A2HS	0.03	0.63 (0.48–0.78)	0.10	0.28	45	38	0.54 (0.38–0.69)	0.63	0.80	17	3	0.41
C4BPA	−0.05	0.69 (0.55–0.83)	<0.01 **	<0.05 *	48	45	0.55 (0.40–0.71)	0.50	0.80	24	14	<0.05 *
C6	−0.13	0.66 (0.51–0.80)	<0.05 *	0.15	41	28	0.57 (0.42–0.73)	0.34	0.80	15	7	0.44
CA1	0.45	0.57 (0.42–0.72)	0.37	0.58	24	17	0.57 (0.42–0.73)	0.34	0.80	15	6	0.97
CD5	0.00	0.57 (0.41–0.73)	0.36	0.58	48	21	0.57 (0.42–0.72)	0.36	0.80	15	7	0.99
HSPA5	0.08	0.57 (0.42–0.73)	0.80	0.94	24	17	0.52 (0.37–0.67)	0.35	0.80	15	7	0.62
IGHG1	−0.21	0.55 (0.40–0.70)	0.78	0.94	31	10	0.52 (0.37–0.67)	0.55	0.80	13	5	0.82
PLS2	−0.03	0.54 (0.39–0.69)	0.58	0.81	24	7	0.57 (0.41–0.72)	0.40	0.80	15	7	0.84
S100A9	−0.02	0.57 (0.42–0.72)	0.33	0.58	34	28	0.51 (0.36–0.66)	0.88	0.94	12	6	0.57
SAP	−0.03	0.72 (0.58–0.85)	<0.01 **	<0.05 *	55	34	0.50 (0.35–0.66)	0.96	0.96	12	5	<0.05 *
SERPINF2	−0.06	0.51 (0.36–0.66)	0.90	0.94	28	17	0.55 (0.39–0.70)	0.56	0.80	21	14	0.75
SPARC	0.00	0.62 (0.47–0.77)	0.13	0.30	41	17	0.54 (0.39–0.69)	0.63	0.80	17	8	0.47
TR	−0.08	0.51 (0.35–0.66)	0.94	0.94	24	21	0.59 (0.45–0.74)	0.21	0.80	15	7	0.41

Abbreviations: AUC, area under the receiver operating curve; CRC, colorectal cancer; 95% CI, 95% confidence interval; IpA, immunome protein array; LC/MRM-MS, liquid chromatography/multiple reaction monitoring–mass spectrometry; *p*-value, apparent *p*-values without any adjustments; *p*-value ^adj^, *p*-value after adjustment for multiple testing by the Benjamini–Hochberg method; Se, sensitivity; Sp, specificity. Protein abbreviations: A1BG, alpha-1B-glycoprotein; A2HS, alpha-2-HS-glycoprotein; C4BPA, C4b-binding protein alpha chain; C6, complement component C6; CA1, carbonic anhydrase 1; CD5, CD5 antigen; HSPA5, 78 kDa glucose-regulated protein; IGHG1, immunoglobulin gamma-1 chain C region; PLS2, plastin-2; S100A9, protein S100-A9; SAP, serum amyloid P-component; SERPINF2, alpha-2-antiplasmin; SPARC, secreted protein acidic and rich in cysteine; TR, transferrin receptor protein 1. Note: *, Significant at a *p*-value of < 0.05; **, significant at a *p*-value of < 0.01.

## Data Availability

The analyzed datasets are not publicly available.

## Supplementary Materials

Supplementary materials can be found at https://www.mdpi.com/1422-0067/22/3/1189/s1. Table S1: Diagnostic performance for markers overlapping between PEA and IpA measurements for detecting CRC cases from participants in screening colonoscopy.
