# Supplementary material for: Comparison of Proteomic Technologies for Blood-Based Detection of Colorectal Cancer
Source: Int J Mol Sci. 2021 Jan 26;22(3):1189. doi: 10.3390/ijms22031189 (PMC7865621; doi:10.3390/ijms22031189)
Supplement: Supplementary file 1 [file ijms-22-01189-s001.pdf]

International Journal of Molecular Sciences

MDPI

Table S1: Diagnostic performance for markers overlapping from PEA and IpA measurements for detecting CRC cases from participants of screening colonoscopy

Marker	Pearson's Correlation Coefficient	Proximity extension assay measurements					Immunome protein array measurements					DeLong p-value for AUC testing
		AUC (95% CI)	p-val	p-val^{adj}	Sn % at 80% Sp	Sn % at 90% Sp	AUC (95% CI)	p-val	p-val^{adj}	Sn % at 80% Sp	Sn % at 90% Sp	
ANXA1	-0.06	0.53 (0.38-0.68)	0.65	0.76	13	5	0.56 (0.41-0.71)	0.43	0.79	15	7	0.38
ARNT	0.08	0.55 (0.44-0.66)	0.37	0.76	13	5	0.56 (0.41-0.71)	0.41	0.79	14	7	0.24
BIRC2	0.10	0.47 (0.37-0.56)	0.52	0.76	13	5	0.58 (0.43-0.73)	0.29	0.79	15	7	0.59
CAIX	0.06	0.51 (0.36-0.66)	0.90	0.92	15	9	0.51 (0.36-0.66)	0.85	0.88	12	5	0.97
CASP3	-0.08	0.53 (0.38-0.69)	0.66	0.76	14	5	0.55 (0.40-0.70)	0.49	0.79	14	7	0.43
CSTB	0.18	0.56 (0.41-0.71)	0.46	0.76	16	5	0.56 (0.41-0.71)	0.41	0.79	14	7	0.27
EGFR	0.06	0.47 (0.32-0.62)	0.69	0.76	15	7	0.52 (0.37-0.67)	0.79	0.85	16	7	0.95
EPCAM	-0.19	0.58 (0.43-0.73)	0.29	0.76	20	10	0.56 (0.41-0.71)	0.42	0.79	15	9	0.86
EPHA2	-0.03	0.54 (0.39-0.69)	0.62	0.76	18	10	0.57 (0.42-0.72)	0.35	0.79	15	6	0.31
EPHB4	0.10	0.58 (0.43-0.73)	0.29	0.76	19	11	0.57 (0.42-0.72)	0.34	0.79	14	7	0.15
ERBB3	0.15	0.53 (0.38-0.68)	0.65	0.76	12	6	0.56 (0.41-0.71)	0.43	0.79	14	7	0.38
FADD	-0.04	0.56 (0.42-0.71)	0.41	0.76	18	7	0.55 (0.41-0.7)	0.48	0.79	18	11	0.27
FAS	-0.12	0.58 (0.44-0.73)	0.27	0.76	19	13	0.55 (0.40-0.70)	0.48	0.79	14	7	0.20
IRAK4	-0.01	0.48 (0.33-0.63)	0.80	0.86	12	4	0.59 (0.44-0.74)	0.25	0.79	15	7	0.53
ITGB6	0.21	0.49 (0.34-0.64)	0.94	0.94	18	10	0.56 (0.41-0.71)	0.41	0.79	13	6	0.61
LYN	-0.10	0.58 (0.44-0.73)	0.27	0.76	19	8	0.58 (0.43-0.73)	0.31	0.81	30	6	0.13
SMAD5	0.04	0.53 (0.38-0.68)	0.69	0.76	16	8	0.58 (0.44-0.73)	0.27	0.79	14	7	0.28
NF2	0.05	0.55 (0.41-0.70)	0.47	0.76	12	5	0.58 (0.43-0.73)	0.31	0.79	15	7	0.22
PRDX1	-0.03	0.53 (0.38-0.68)	0.66	0.76	13	5	0.49 (0.34-0.64)	0.94	0.94	19	10	0.81
SPARC	0.28	0.51 (0.36-0.66)	0.90	0.92	12	6	0.54 (0.39-0.69)	0.63	0.79	17	8	0.67
TCL1A	-0.11	0.57 (0.42-0.72)	0.35	0.76	18	9	0.54 (0.39-0.69)	0.63	0.79	14	6	0.32
TGFR2	0.09	0.54(0.39-0.69)	0.58	0.76	15	7	0.54 (0.39-0.69)	0.63	0.79	14	6	0.47
TRAF2	0.02	0.57 (0.42-0.72)	0.35	0.76	16	7	0.52 (0.37-0.67)	0.82	0.86	18	10	0.62
ZBTB16	0.12	0.54 (0.39-0.69)	0.64	0.76	14	6	0.56 (0.41-0.71)	0.41	0.79	16	7	0.81

Abbreviations: AUC–area under the receiver operating curve; CRC–colorectal cancer; 95% CI–95% confidence interval; IpA– immunome protein array; PEA–proximity extension assay; p-val–apparent p-values without any adjustments; p-val^{adj}–p-value after adjustment for multiple testing by Benjamini Hochberg method; Se–sensitivity; Sp–specificity. All proteins abbreviations: ANXA1– Annexin A1; ARNT– Aryl hydrocarbon receptor nuclear translocator; BIRC2– Baculoviral IAP repeat-containing protein 2; CAIX– Carbonic anhydrase IX; CASP3– Caspase-3; CSTB– Cystatin-B; EGFR– Epidermal growth factor receptor;; EPCAM– Epithelial cell adhesion molecule; EPHA2– Ephrin type-A receptor 2; EPHB4– Ephrin type-B receptor 4; ERBB3– Receptor tyrosine-protein kinase erbB-3; FADD– FAS-associated death domain protein; FAS– Tumor necrosis factor receptor superfamily member 6; IRAK4– Interleukin-1 receptor-associated kinase 4; ITGB6– Integrin beta-6; LYN– Tyrosine-protein kinase Lyn; NF2– Merlin; PRDX1– Peroxiredoxin-1; SMAD5– Mothers against decapentaplegic homolog 5; SPARC– Protein SPARC; TCL1A– T-cell leukemia / lymphoma protein 1A; TGFR2– TGF-beta receptor type-2; TRAF2– TNF receptor-associated factor 2; ZBTB16– Zinc finger and BTB domain-containing protein 16.
